# Functional crosstalk between the lung and eye: a new frontier in chronic lung diseases

**DOI:** 10.1007/s00204-025-04136-7

**Published:** 2025-07-27

**Authors:** Hannah Taylor Lee, Hudson C. Taylor-Blair, Dinesh Kumar Chellappan, Gabriele De Rubis, Keshav Raj Paudel, Brian G. Oliver, Kamal Dua, Stewart Yeung

**Affiliations:** 1 Discipline of Pharmacy, Graduate School of Health, University of Technology Sydney, Ultimo, NSW 2007 Australia; 2 Faculty of Health, Australian Research Centre in Complementary and Integrative Medicine, University of Technology Sydney, Ultimo, NSW 2007 Australia; 3 School of Life Sciences, Faculty of Science, University of Technology Sydney, Ultimo, NSW 2007 Australia; 4 Centre for Inflammation, Faculty of Science, School of Life Sciences, Centenary Institute and University of Technology Sydney, Sydney, NSW 2007 Australia; 5 Woolcock Institute of Medical Research, Macquarie University, Sydney, NSW Australia; 6 Department of Life Sciences, School of Pharmacy, IMU University, Bukit Jalil, Kuala Lumpur, Malaysia; 7 NICM Health Research Institute, Western Sydney University, Westmead, NSW 2145 Australia; 8 Uttaranchal Institute of Pharmaceutical Sciences, Uttaranchal University, Dehradun, India; 9 Centre of Medical and Bio-Allied Health Sciences Research, Ajman University, Ajman, United Arab Emirates

**Keywords:** Lung–eye, Inflammation, Infections, Respiratory diseases, Crosstalk

## Abstract

Respiratory diseases are among the main causes of morbidity and mortality worldwide, encompassing a wide array of illnesses. Among these diseases, including acute lung injury, chronic obstructive pulmonary disease (COPD), asthma, pulmonary fibrosis, obstructive sleep apnoea (OSA), and pathogenic infections, the immune system plays a significant role in whole-body pathophysiology. These occurrences have been recognised to affect the ocular system, bringing about the novel idea of the lung–eye axis with emerging literature highlighting the fundamental connection of exacerbation between systems. Prior literature has recognised axial activity across systems, the gut and eye, where gut microbiota has an indicated correlation with the ocular environment. In addition, crosstalk has been hypothesized in a brain–lung axis via neurological anatomy, immune mechanisms and microbial pathways. Such cascades offer foundation for the lung–eye axis, supporting the potential for a correlative relationship between the ocular and respiratory system through anatomical, mucosal and inflammatory crosstalk. Although in its infancy, the interconnection between ocular and respiratory systems has been considered in the development of chronic diseases. Amid chronic diseases, COPD, OSA and glaucoma exhibit underlying mechanisms, incorporating hypoxia, oxidative stress and vascular dysfunction, postulating dual system pathophysiology. Finally, potential biomarkers are proposed following pathophysiological mechanism exploration, with an advocation for longitudinal studies in future. The current review proposes a novel axis in the field of lung diseases and aims to provide significant insights for respiratory and ocular clinicians, in addition to translational researchers, paving a new path for understanding systemic disease and treatment modality.

## Introduction

Chronic respiratory diseases are among the main causes of mortality and are the leading causes of morbidity worldwide (Mehta et al. 2020; Viegi et al. 2020; Xie et al. 2020). In 2021, the World Health Organisation reported, that respiratory diseases represented 50% of the top 10 global causes of death, with illnesses, including COVID-19, lower respiratory infections, chronic obstructive pulmonary disease (COPD), and lung cancer. The susceptibility of the human respiratory system can be exploited by various detrimental factors, among them, environmental insults, and occupational and behavioural inhalation hazards, namely, smoking (Dharwal et al. 2020; Labaki & Han 2020; Taylor-Blair et al. 2024).

Pulmonary conditions constitute a diverse expansion of pathologies related to respiratory diseases, varying from acute illnesses, such as influenza and coronaviruses, to recurring conditions, including COPD, obstructive sleep apnoea (OSA), asthma, interstitial lung disease, and even cancers (Bhat et al. 2024; Haysom‐McDowell et al. 2025; Labaki & Han 2020; Patel et al. 2024). Despite the prevalence of chronic respiratory disease, the heterogeneity of clinical presentations and the often complex underlying pathophysiology has resulted in an incomplete understanding of respective disease mechanisms and poor prognoses (Labaki & Han 2020; Tan et al. 2022; Williams et al. 2020).

Bidirectional relationships between organs and systems have become a recent focus of research concerning the systemic implications of local diseases (Campagnoli et al. 2023; Thapa et al. 2024; Wang et al. 2023), allowing for the unveiling of underlying mechanisms and communications between systems. The recent findings of an eye–gut and gut–lung axis has brought forward the notion of the roles played by microbes in systemic health through the use of circulating inflammatory mediators (Campagnoli et al. 2023; Wang et al. 2023). An altered gut microbiome has been linked with modifications in the lung immune responses and airway homeostasis, highlighting the large interplay between systems within the human body (Dang & Marsland 2019; Paudel et al. 2020; Thapa et al. 2024). The recent advancement of a brain–lung axis further supports this idea with an interconnection between systems, including neuroanatomical, endocrine, immune and micro-organism pathways (Li et al. 2023). Systemic hypoxia and oxidative stress within lung diseases, as seen in COPD, has been documented in intestinal dysfunction, prompting the role of axes in health and the systemic exposure of lung disease (Wang et al. 2023).

Building on the concept of the systemic axis, the suggestion of a “lung–eye axis” has emerged within the literature following the discovery of altered retina circulation in pulmonary arterial hypertension (Nickel et al. 2020). This notion has offered innovative revelations in both direct and indirect mechanisms for respiratory pathogens and/or inflammatory responses affecting the ocular region (Allam et al. 2024). Importantly, a bidirectional relationship has been proposed via the cross talking of the two systems, a crucial idea when considering the constant exposure of the eye to external environments (Allam et al. 2024). With this new concept, the ocular system may serve as a novel indicator of systemic health and a pivotal biomarker for respiratory diseases (Allam et al. 2024). The bidirectionality of the lung–eye axis offers a glimpse into the expansion of pathogens from the respiratory system, elucidating the mechanisms by which such pathogens disseminate and transmit to other tissues (Belser et al. 2013).

This review aims to investigate the pathophysiological connections between the lung and eye in each direction of pathogenic spread by investigating the internal transmission of communicable diseases, such as tuberculosis and coronaviruses via anatomical, vascular, and immunological processes. Noncommunicable disease interplay is explored, ranging from ocular and lung cancer, to COPD, OSA, and glaucoma, emphasizing connections between distant systems and the role of the lung–eye axis. Immunological and inflammatory mechanisms are explored by analysing anatomical, physiological, and pathogenic processes involved in the interplay between the systems. Furthermore, this review aims to offer a comprehensive understanding of the proposed lung–eye axis and attempts to enhance knowledge of systemic disease interactions.

## Respiratory and ocular immunity

Mucosal immune systems and regulations have had great attention within the literature, albeit in isolated mucosal sites, with dysregulation of such immunity suggested to play central roles underpinning a meriad of chronic diseases ranging from asthma and COPD, to dry eye disease and uveitis (Lee et al. 2014; Tulic et al. 2016). With frequent discoveries positing the integration of tissues, cells and immune responses through a network, the parallels between the respiratory and ocular mucosal immune systems may offer a new realm of communication.

Comprising primarily of the conjunctiva and cornea, the ocular surface is home to an ecosystem of micro-organisms. This is now recognised despite previously being considered a sterile environment (Allam et al. 2024). The ocular microbiome, inhabiting the ocular surface, contains a highly variable and diverse range of different micro-organisms; bacteria are the most abundant, although viruses, fungi, and protozoa are also known to inhabit the surface (Aragona et al. 2021). This microbial community serves important roles in pathogen defence the ocular mucosal immune system, known as the eye-associated lymphoid tissue (EALT), and comprising of the conjunctiva-associated lymphoid tissue (CALT) and the lacrimal drainage-associated lymphoid tissue (LDALT) (Knop & Knop 2005).

Bacterial genera identified have included *Staphylococcus, Streptococcus, Cornyebacterium, Bifidobacterium, Moraxella, Dolosigranulum,* and *Propionibacterium* (Aragona et al. 2021). Although recent research has improved our understanding, the typical homeostatic composition of the ocular surface’s microbial community has yet to be elucidated (Aragona et al. 2021). Perturbations in the ocular microbiome can occur as sequalae of a range of diseases, such as dry eye, ocular allergies, conjunctival lymphoma, Stevens–Johnson syndrome, and *Chlamydia trachomatis* infection (Aragona et al. 2021). Considering the ocular tropism of respiratory viruses, it is possible that respiratory disease or complications could alter the ocular microbiome (Belser et al. 2013). This presents itself as a novel area of research requiring further investigation as current evidence regarding the ‘lung–eye axis’ is sparse.

Similar to the newer discovery of the ocular microbiota, the lungs were once thought to be devoid of bacteria; however, there is now compelling evidence of the lung and the respiratory system containing a diverse community of micro-organisms (Dickson et al. 2016). The lung microbiome is only now becoming recognised as an important contributor to respiratory immune function, paralleling the ocular system through its imperative mucosa-associated lymphoid tissue (MALT). Like the EALT, the respiratory MALT is comprised of three structures, the bronchus-associated lymphoid tissue, lacrimal-associated lymphoid tissue and the nasal-associated lymphoid tissue (Debertin et al. 2006). An important note is the anatomical connection between the LDALT and the LALT, whereby tears secreted in the LALT flow across the eye and into the LDALT, allowing for antigen and possibly local microbial products and subsequent signals to interact and between systems. The upper respiratory tract has large bacterial diversity (Shilts et al. 2016). The upper respiratory tract has a distinct microbiome compared to that of the oral cavity (Bassis et al. 2014), including nasal commensal bacteria comprising *Staphylococcus aureus* and *Moraxella catarrhalis* (Pettigrew et al. 2008). Furthermore, commensal bacteria in the upper respiratory tract can have pathogenic roles, as seen in bacterial pneumonia, when reaching other areas of the body, in particular, the lower respiratory tract (Stearns et al. 2015). The lower respiratory tract microbiome is more akin to the oral microbiome, with common commensal bacteria genera, including *Prevotella, Veillonella* and *Streptococcus* (Huffnagle et al. 2017).

The imperative antibody, secretary immunoglobin A (sIgA), has been found to be pivotal in the maintenance of both EALT and MALT regulation through the neutralisation of invading pathogens and the stimulation of anti-inflammatory responses. Within the ocular surface, sIgA is secreted in the lacrimal glands and is secreted by the lacrimal glands and regulated by the ocular microbiota to promote interleukin-10 (IL-10) production as well as dendritic cell modulation (Allam et al. 2024). Likewise, IgA responses within the lung environment are governed by lung microbiota through toll-like receptor activation and, analogous to the eye, dendritic cell mediation (Allam et al. 2024). Dendritic cells in both the BALT and the EALT (conjunctiva and lacrimal glands) express surface antibodies, including CD103, enabling the endorsement of IgA class switching through T cell pathways. This expression suggests that both the lung and eye sIgA concentrations are dependent upon local microbes and have strong involvement of immunity in both systems, offering a potential way for crosstalk within a connected immune system. Potential mucosal communication has already been stated by Kim et al*. *via eyedrop inoculation of mice with 1 ug H1N1 (influenza) + 10 ug poly(I:C) was effective at inducing a strong immune reaction within the respiratory immune system compared with control (PBS administered) (Kim et al. 2015). Following ocular administration, mice demonstration significantly upregulated (*P* < 0.001) IgA antibodies in the lungs, measured through bronchoalveolar lavage fluid (no further quantitative data given) (Kim et al. 2015). The observed mucosal IgA activity was congruent with prominent viral clearance, being significantly reduced 4 day postinfection (DPI) (*P* < 0.05), and no detectable virus in the lungs 7 DPI (*P* < 0.001), compared to PBS administrated groups (Kim et al. 2015). Further cementing the bidirectionality of mucosal communication, intranasal immunisation of the with DNP-conjugated antigen, in rats, resulted in alterations of tear IgA concentrations (Carr et al. 1996). Throughout the measured timepoints, IgA concentrations continued to be higher than other forms of administration, ocular-topical and gastrointestinal, peaking 10 DPI with a fold increase of 1.4 (Carr et al. 1996). These results demonstrate a robust increase in ocular immune response and depict prominent crosstalk from the NALT to the EALT, further consolidating the communication between the lung and eye.

Alluding further to the connection between the ocular and lung microbiome is the dissemination of micro-organisms, along with their products and immune cells, pioneers the communication between the lung and the eye (Allam et al. 2024). The imbalance within the ocular microbiome, ocular dysbiosis, can result in chronic inflammation along with diseases, such as dry eye syndrome and uveitis (Ozkan et al. 2023). The resulting inflammatory mediators are not restrained to the eye but can travel through systemic circulation and potentially into the respiratory tract, culminating in respiratory inflammation. The idea of such respiratory implications has been supported with the detection of systemic inflammation in patients with a history of anterior uveitis (Huhtinen et al. 2002). The study found evidence of persistent systemic immune activation in patients with previous anterior uveitis, yet without current ocular inflammation, compared to healthy controls (Huhtinen et al. 2002). Following administration of bacterial lipopolysaccharides (10 ng/mL and 1000 ng/mL) to blood samples from both healthy controls and patients, it was found that even in the absence of current uveitis, the systemic immune system of the patients exhibited a primed innate response, producing significantly greater amounts (*P* = 0.012) of tumour necrosis factor α (TNFα) upon microbial stimulus (Huhtinen et al. 2002). In addition, patients displayed significantly more high-sensitivity C-reactive protein (hs-CRP) than control (1.59 ug/mL vs 0.81 ug/mL), an important observation due to the protein usually being produced in response to inflammatory and infectious conditions (Huhtinen et al. 2002).

The potential for ocular inflammatory mediators to spill into systemic circulation, a key mechanism required for a lung–eye axis, is further posited by the rat model of endotoxin-induced uveitis (Hoekzema et al. 1991). Intravitreal administration, directly into a single eye, of IL-6 was found to influence both the ocular and systemic environment, where injection into one eye resulted in concurrent protein leakage through IL-6 diffusion into the blood stream and subsequent homing to the other eyes vascular bed (Hoekzema et al. 1991). Importantly, aqueous humour found to have ~ 10 × higher IL-6 levels than serum, advocating to a significant immune response from the eye that can potentially contribute to a greater systemic, and possibly respiratory, inflammatory milieu (Hoekzema et al. 1991). Unfortunately, due to gaps in the literature, there is minimal research investigating the systemic spillover of ocular diseases, and no present studies found investigating the potential of respiratory infiltration of ocular spillover, concrete mechanisms are yet to be determined. However, the existence of the aforementioned studies suggest the potential of such infiltration and highlight the interconnections amongst immune pathways.

There is growing evidence regarding the importance of the microbiome in a diverse range of diseases. Changes to the respiratory microbiome are shown to be intrinsically related to clinical perturbations in a range of chronic lung diseases, including asthma (Huang et al. 2011), bronchiectasis (Rogers et al. 2014) and idiopathic pulmonary fibrosis (Molyneaux et al. 2014). Due to the crosstalk between the eye and the lung having the physical connection via the nasolacrimal duct, it is possible that changes in either the ocular or respiratory microbiome could cause diseases, with the example of asthma development resulting from ocular dysbiosis (Allam et al. 2024; McDermott 2013). Future work could investigate if associations exist between other chronic eye diseases and changes in the respiratory microbiome. Furthermore, these connections highlight how novel microbiome-altering therapeutics showing promising results with other organ microbiome such as gut microbiota may improve chronic eye diseases (Elwakil et al. 2023).

## Ocular–respiratory connection: exploring the role of the nasolacrimal duct

**Fig. 1:**
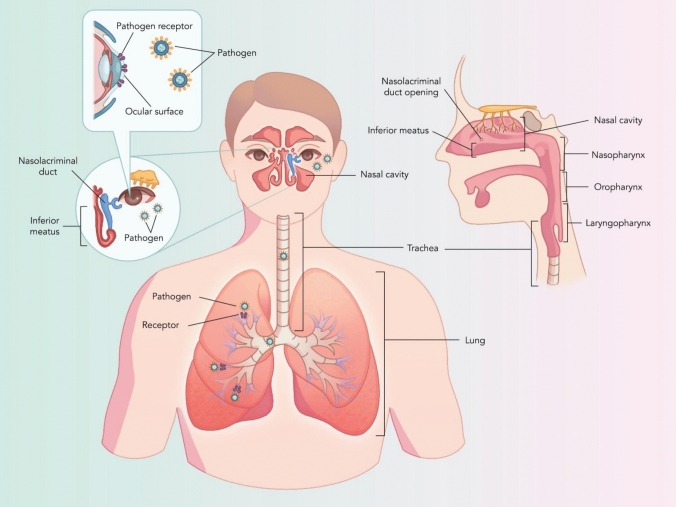
Anatomical linkage between the ocular and respiratory system. The nasolacrimal duct acts as the physical connection between the ocular surface and the respiratory tract, demonstrating a probable route for immune, microbial and pathogen interactions in the lung–eye axis. The image depicts the drainage route from the ocular surface to the inferior meatus of the nasal cavity and descension of pathogens into the respiratory system

The nasolacrimal duct (NLD) acts as the physical bridge between the ocular and respiratory systems, connecting the eye-associated lymphoid tissue and the nasal-associated lymphoid tissue, both of which are pivotal components of the mucosal immune system (Belser et al. 2013; Chentoufi et al. 2010). Tears and pathogens are absorbed from the ocular surface into the lacrimal punctum and circulates through the canalicular, within the eyelids, to the ampulla, or fluid reservoir (Ducker & Rivera 2023). Secretions then drain into the lacrimal sac, where the lower half of the lacrimal system begins and flows through the NLD, into the inferior meatus of the nasal cavity, and subsequently into the upper respiratory system (Fig. 1) (Ducker & Rivera 2023). The absorption of tear components, and other contaminants within the film, occurs in the microvilli-lined epithelial cells of the NLD (Belser et al. 2013). Finally, any pathogenic substance descends through to the trachea and into the lower respiratory system and lungs (Fig. 1).

Instances of NLD transmission have been documented in human coronavirus (HKU1), respiratory syncytial virus (RSV) and adenovirus genomes using dacryocystorhinostomy samples (Yanık et al. 2021). Yanik et al. found that, even in cases of NLD obstruction, virions continued to enter the inferior meatus, thus the respiratory system (Yanık et al. 2021). The results indicated that 8.3% of patients had viral genetic material in the proximal duct mucosa, and 20.8% had viral genetic material in the nasal cavity and inferior meatus swabs. This highlights that viral expansion can still occur from the canaliculi, through the duct, and into the inferior meatus despite an obstructed. Interestingly, the HKU1 viral genome was not detected in the nasal swab of the inferior meatus, only the dacryocystorhinostomy samples. This is likely due to the presence of the functional transmembrane serine protease 2 (TMPRSS2) on the ocular surface, allowing for HKU1 entrance (Saunders et al. 2023).

The movement of eye commensal translocation through the NLD, to the lung, is mirrored by other viruses (Allam et al. 2024; Vieira et al. 2018). This was proven possible in primates after administering five rhesus macaques with an infectious dose (1 million infectious units) of severe acute respiratory syndrome coronavirus 2 via gastric, tracheal, or conjunctival routes (Deng et al. 2020). Results from the investigation yielded the presence of viral RNA in nasal and throat swabs from conjunctival deposit. Furthermore, real-time quantitative polymerase chain reaction (RT-qPCR) revealed a high viral load in the nasolacrimal system (Belser et al. 2013). Viral RNA was detected in the nasolacrimal tissues, including the lacrimal gland, nasal cavity, nasal mucosa, and throat, along with the optic nerve and conjunctiva, demonstrating dual system susceptibility (Deng et al. 2020). Accumulation of inflammation and fluid was documented in spaces between the alveoli of the monkeys, confirming the lung impact of ocular inoculation (Deng et al. 2020). With the viral RNA detection being found throughout the upper respiratory tract, the left lobe of the lung, as well as the nasolacrimal tissues and conjunctiva, the concept of pathogenic transfer through the NLD has been strongly supported (Deng et al. 2020). These outcomes offer potential for innovative treatment opportunities for targeting the pulmonary system through less invasive approaches. For example, Timolol is a treatment for glaucoma administered via eye drops, yet has been reported to have systemic and pulmonary side effects (Patel et al. 2015), proposing the idea of ocular drug delivery exploration for non-invasive respiratory treatments.

The NLD plays a pivotal role in the physical linkage between the ocular and respiratory systems and facilitates more than tear drainage but the transmission of pathogens. With viral detection throughout the ocular and lacrimal system, the potential exposure of the environment to the respiratory system is depicted and demands further investigation for the safeguarding of both systems. Even during NLD obstruction, the route is still viable for the transmission of pathogens, removing misconceptions about obstructive mechanisms. The implications for understanding these mechanisms can yield progress in both the understanding of pathogen and inflammation movement as well as novel treatment options.

## The respiratory to ocular system

In the nasolacrimal system, two crucial valves exist to intercept tear reflux, the backward passage of tears and constituents from the NLD into the eye: the valve of Hasner and the valve of Rosenmuller. Despite one-way ensuring mechanisms, the force of coughing enables secretions from the nasopharyngeal tract to penetrate the conjunctival sac via the NLD (Yener 2021). This infiltration process has been depicted in the conjunctival manifestations of SARS-CoV-2 (Ma et al. 2020). Individuals with systemic symptoms, such as coughing, were at a greater risk of developing ocular symptoms, with 22.7% of patients in a recent study reporting ocular symptoms, the primary being increased conjunctival discharge (Ma et al. 2020). Such instances have been termed “nasolacrimal reflux” and have been documented in adults and children with a history of acute or chronic conjunctivitis due to vigorous nose blowing (Chang et al. 2006). With high frequency coughing being a commonality across various respiratory illnesses, the nasolacrimal reflux may yield a pivotal role in the conversion of local respiratory illness to systemic infection within the ocular system (Li et al. 2023).

The bidirectionality of this anatomical link has not been extensively investigated amongst current literature but cannot be overlooked in its pathogenic-transmission capability. The potential for comprehending such transmission may help disease management via early diagnosis, systemic spread mechanisms, and inflammatory communication between systems. Finally, recognising this interaction can extend to the understanding of respiratory noncommunicable and inflammatory diseases and their impact on the ocular system, allowing for early intervention, such as clinical tear draining and improved prognosis.

## Viruses

### Incidence of invasion of the respiratory system through the ocular surface

Communication between the ocular and respiratory systems extends to a variety of shared receptors, allowing for pathogen entrance into both systems (Belser et al. 2013). By 2013, literature only began to elucidate this relationship in a series of respiratory viruses, including adenoviruses, coronaviruses, respiratory syncytial virus, and rhinoviruses, all of which have entrance receptors within the ocular system (Belser et al. 2013). By 2022, further investigation evidenced the presence of viral receptors on the ocular tissues and found them to parallel that of the respiratory system, opening the eye to similar infectious potential (Petrillo et al. 2022) Viral pathogens are transmitted through the droplets of those infected via coughing, sneezing, or contact with the ocular surface, nose or mouth, all of which are characterised by a mucous membrane (Allam et al. 2024). The exposure of the conjunctiva to the external environment has frequently been subject to pathogen and pollutant invasion (Alves et al. 2023). Despite the commonality of acute upper respiratory tract infections contracted via this route, advancement to lower respiratory organs does occur (Belser et al. 2013). The dispersion of receptors throughout the nasolacrimal system, conjunctiva, and cornea contribute to host invasion of viral particles. Commonly occurring ocular manifestations of respiratory viruses include conjunctivitis and keratitis, which frequently occur in cases of infection with avian influenza viruses (H7), human adenovirus D, monkeypox and SARS-CoV-2 (Allam et al. 2024; Taha et al. 2022).

### Influenza viruses and the ocular surface

The human nasal mucosa and trachea are abundant in alpha2-6-linked epithelial sialic acid (SA) glycoprotein receptors, with the lower respiratory tract being dominated by alpha2-3-linked sialic acids (Belser et al. 2013). Such expression is mirrored by the ocular system, where the cornea and conjunctiva present an abundance of alpha2-3 SAs and the NLD has a distribution of both glycoprotein subtypes (Belser et al. 2013). With this expression, viral entry can be observed and has been documented in zoonotic influenza viruses (Belser et al. 2013). The receptor presentation may allow for prominent invasion and replication of highly pathogenic mutated influenza A variants within the ocular surface (Belser et al. 2011). Significant subtypes include H7N7, the strain linked to conjunctivitis in the Netherland’s 2003 outbreak (Olofsson et al. 2005), H5N1, associated with the ocular infections of the Hong Kong 1997 outbreak (Olofsson et al. 2005), and H7N3, causing ocular symptoms in the Canadian 2004 outbreak (Belser et al. 2011). Importantly, differential cytokine and immune signalling in ocular-targeting subtypes, such as H7N7, has been recognised compared with that of respiratory targeting subtypes. Belser and colleagues made this evident when comparing ocular cells (corneal epithelial, conjunctival epithelial and trabecular meshwork cells) with bronchial epithelial cells (Belser et al. 2011). Following H7 viral inoculation, significant stimulation of nuclear factor kappa B (NF-κB) signalling was found in ocular cell types and is contrasted by the downregulation of related genes in respiratory cells, indicating a selective localised response to the subtype (Belser et al. 2011, 2013).

Of note, mutations in respiratory viral subtypes have been suggested to play a role in ocular surface infection.

Specifically, the substitution of lysine at position 627 in the polymerase basic 2 protein, seen in H7N7 and H5N1 among others, has been postulated to enhance replicative ability of the virus in the human ocular system (Creager et al. 2018; Yang et al. 2012). In addition, replacement of alanine with threonine via glycosylation at position 125 of hemagglutinin, is observed in H7N7, and is one of the binding proteins responsible for the attachment to host cell sialic acids (Creager et al. 2018). The glycosylation is hypothesized to increase binding affinity to alpha2-3 linked SAs on the ocular surface, and may demonstrate an evolutionary adaptation for cross-species infection (Creager et al. 2018). Despite these propositions, a definitive answer to the ocular tropism of respiratory viruses has yet to be established; however, due to the high expression of alpha2-3-linked SAs on the ocular surface, the eye is inherently more susceptible to avian influenza strains compared with the presence of alpha2-6-linked receptors within the upper respiratory tract. Furthermore, avian strains can result in greater pathogenicity due to the location of alpha2-3-linked receptors in the lower respiratory system, namely, the lungs (Van Riel et al. 2006), highlighting a need for understanding the role of ocular entrance.

Due to a lack of rigorous investigation surrounding viral entrance through the eye, individuals, including healthcare workers, are often less adherent to eye protection usage. This hypothesis was suggested in 1986 by Gala and colleagues, who found that the use of eye goggles resulted in a decline of RSV infections among both children and adults (Gala et al. 1986). Following 3 weeks of goggle usage, 5% of adults and 6% of children accumulated infection, yet, without eye protection, 34% of adults and 43% of children became infected, demonstrating a significant impact of eye protection in preventing RSV transmission (Gala et al. 1986). This incidence of eye inoculation was further documented in human influenza viruses and RSV with significant emphasis on conjunctival, corneal, and nasolacrimal epithelia receptor sharing (Bischoff et al. 2011; Mermel 2018).

Using a live attenuated influenza viral formulation containing two influenza A strains (H1N1 and H3N2) and one influenza B strain, engineered to replicate at cool temperatures, Bischoff and colleagues exposed 6 groups of healthy individuals to viral particles via a vibrating-orifice aerosol generator (Bischoff et al. 2011). The 6 groups included (1) no protection, (2) ocular-only exposure, (3) surgical mask no eye protection, (4) surgical mask with non-vented Z87 Uvex Goggles, (5) N95 respirator without eye protection, and (6) N95 respirator with non-vented Z87 Uvex Goggles. The study concluded that ocular protection is essential for the prevention of the ocular transmission of the live attenuated influenza virus strains tested (Bischoff et al. 2011). This was deduced through results demonstrating only 20% of participants in group 6, with both N95 respirator and ocular protection, having detected influenza via nasal wash viral culture (Bischoff et al. 2011). In contrast, group 5, N95 respirator without ocular protection, had a 60% viral detection rate. The discrepancy between groups 5 and 6 suggests the potential of a significant increase in influenza transmission when ocular preventions are lacking.

To further emphasize the importance of ocular protection, the variations between groups 1 and 2 suggest a strong association with group 2 having the lowest amount (5 copies) of detected RNA through RT-qPCR extracted from nasal wash samples (Bischoff et al. 2011). These outcomes advocate, not only that ocular protection adds some amount of protection, but the probable anatomical transmission of pathogens through the NLD into the nasopharynx (Bischoff et al. 2011). Bischoff et al*.* emphasize the rapid transfer of viral particles with influenza RNA detected in the nasopharynx in group 2 a mere 30 min following inoculation (Bischoff et al. 2011). This observation supports the concept of the lung–eye axis by highlighting the conduit potential of the NLD from the ocular system to the respiratory system.

Here the reciprocal potential for tropism amongst the eye and lung systems is outlined amongst the influenza viral strains, both human and avian. With the literature elucidating such mechanisms being sparse and experimental evidence in its infancy, further studies should look to uncover the influence of receptor distribution amongst the ocular and respiratory environment and its influence on respiratory exposure.

### Severe acute respiratory syndrome coronavirus (SARS-CoV) family

The severe acute respiratory syndrome coronavirus 2 (SARS-CoV-2), like its predecessor, SARS-CoV, responsible for the 2002 SARS pandemic, enters host cells via the angiotensin converting enzyme 2 (ACE2) and is then primed by transmembrane serine protease 2 (TMPRSS2), a protease shared by the influenza virus for activation (Glowacka et al. 2011). With respiratory symptoms of coughing and breathing difficulty, evidence has unveiled the presence of ACE2 in nasal epithelial cells, lung cells, and bronchial epithelial cells using immunohistochemical analysis (Zhou et al. 2020). However, the lung is not the only organ impacted by SARS-CoV infections, as published research has suggested SARS-CoV impacts other organs, including the eyes and the skin (Baj et al. 2022; Imran et al. 2023). Single-cell RNA sequencing results revealed the ACE2 receptor has the greatest expression within the sinonasal cavity, allowing for the speculation of respiratory system entrance through the NLD (Ortiz et al. 2020). Through immunohistochemical analysis, the presence of both TMPRSS2 and ACE2 has been revealed on conjunctival epithelial cells, corneal cells, and the limbus, yet ocular protection is still controversial (Belser et al. 2013) (Collin et al. 2021) (Byambasuren et al. 2021; Roehrich et al. 2020). The assumption of ocular transmission is further supported by ocular symptoms being initially implicated in patients affected by SARS-CoV-2 (Ozturker 2021; Qu et al. 2021a, b). Conjunctivitis has been a common initial, or sole, symptom (Qu et al. 2021a, b). Qu et al. found that > 23% of COVID-19 patients suffering from the ocular conditions, exhibited symptoms ranging from conjunctival hyperaemia and watery secretions, to severe symptoms, including optic neuritis and retinal vascular diseases (Qu et al. 2021a, b).

Li and colleagues tested for the expression of ACE2 receptors in human conjunctival tissue to determine expression variations in inflammatory ocular diseases, including conjunctivitis, conjunctival nevi, conjunctival papilloma, conjunctival cyst and conjunctival polyps, against a normal control (Li et al. 2020). Diseased human conjunctival epithelial samples revealed a statistically significant overexpression of ACE2 mRNA (*P* < 0.001) in conjunctival tissues compared with the non-inflamed, evidenced by RT-qPCR of conjunctival samples (Li et al. 2020). Significant increases were seen further in protein levels of ACE2 in the diseased conjunctival tissues vs the normal tissues (*P* < 0.001) through immunohistochemistry and Western blotting, demonstrating a functional response to mirroring that of a diseased respiratory system, suggesting higher potential for infection shared across both systems when diseased (Li et al. 2020; Pinto et al. 2020). The discovery supports the hypothesis and findings from Collin and colleagues, that inflammation can upregulate ACE2 expression and may increase the susceptibility of the conjunctiva to SARS-CoV-2 and other pathogens using the ACE2 receptor for entry (Collin et al. 2021; Li et al. 2020). Furthermore, detection of the virus has still resulted in positive conjunctival swabs even in the absence of ocular symptoms, indicating the molecular attraction of the ocular surface to the SARS entry proteins (Qu et al. 2021a, b).

To address uncertainty surrounding the systemic spread of SARS-CoV-2, Jeong et al*.* infected transgenic mice, that express the human ACE2 receptor, through multiple inoculation routes, including intranasal (IN), intratracheal (IT), intravenous (IV), and intracerebral (IC) (Jeong et al. 2022). After undertaking RT-qPCR, histological, and immunofluorescence staining, results demonstrated a unidirectional spread of pathogens to the respiratory system compared to wild-type control (Jeong et al. 2022). Retinal inflammation, followed by increased retinal thickness, was found following IN inoculation with immunofluorescence analysis demonstrating immune cell infiltration of neutrophils and T cells, as evidenced by the Gr-1 (neutrophil marker) and CD3 (T cell marker) 6 DPI (Jeong et al. 2022). An increase in pro-inflammatory cytokines and chemokines was found, among them, granulocyte colony-stimulating factor (G-CSF), interferon gamma-inducible protein 10 (IP-10), monocyte chemoattractant protein-1 (MCP-1), macrophage–inflammatory protein-2 (MIP-2), (interleukin-6) IL-6 and IL-12, depicting a strong immune response. Importantly, this elevation was determined significantly higher in the eyes and the brain when compared to the lungs (Jeong et al. 2022). It was noted that viral titre within the eyes was as abundant as in the lungs, indicating the efficiency of viral dissemination from the respiratory tract, and an augmented immune response within the ocular system. Intratracheal administration, directly into the lungs, facilitated dissemination of viral particles from the lungs to the brain and ocular system via the trigeminal nerve (TN) and optic nerve (ON), underscoring a new route for pathogenic spread between systems (Jeong et al. 2022). The observation of nerve dissemination was confirmed by IC inoculation, where viral RNA was detected in the ON and TN, eye globes and brain with comparably low levels in the lungs (Jeong et al. 2022). Due to the low RNA expression in the lungs, it can be speculated that the direct inoculation of the central nervous system resulted in indirect dissemination of the virus into the lungs, implicating the brain–lung axis.

At both the molecular and clinical levels, a tropism is exhibited by the coronavirus family, highlighting the possibility for ocular entrance. With the pathogenic family being vastly transmissive and having caused multiple pandemics, the susceptibility of the ocular system can bring to light improved transmission-preventing protocols. The presence of both ACE2 and TMPRSS2 on the ocular surface epithelium supports the idea of independent viral replication within the ocular surface and its role as a point of viral entry, highlighting the exploitation of the ocular mucosal immune system. Together, the above findings promote the importance of ocular swabs for coronavirus detection and emphasizes the necessity of understanding viral molecular targets.

## Bacteria

### Dissemination of bacteria from the lung to the eye

Dissemination through the blood, hematogenous spread, has become the most agreed upon mechanism by which bacterial transmission occurs between the lung and eye, as seen in ocular manifestations of *Mycobacterium tuberculosis* (Dua et al. 2006; Kansal et al. 2017; Priebe et al. 2004; Thayil et al. 2011). Bacterial invasion results in pulmonary tuberculosis when inhaled via aerosols, yet the infection is not confined to the respiratory system and attacks other organs of the body in 20% of cases (Abdisamadov & Tursunov 2020). The eye is infected in 3.5–5.1% of immunocompetent patients with incidences of ocular invasion increasing in immunocompromised individuals (Abdisamadov & Tursunov 2020). Because of the frequent asymptomatic or latent nature of the ocular disease, paired with a deficiency of investigational techniques, the pathogenesis has been shrouded in ambiguity in the past, yet has garnered increasing interest.

### Mycobacterium tuberculosis

Aerosols containing *M. tuberculosis* (Mtb) inhaled into the lung are engulfed by alveolar macrophages, resulting in the attraction of dendritic cells and more colonizing macrophages (Basu et al. 2020). The guinea pig model is considered a viable model for human ocular pathologies due to both structural, and functional similarities including a resembling inflammatory responses that convey histopathological features congruent to that of a human response (Rao et al. 2009). Furthermore, in proposing its use in the context of the lung–eye axis, guinea pig lungs develop necrotic granulomas and the associated hypoxia upon exposure to bacteria as well as paralleling the immune modulation, tissue remodelling, and angiogenesis of a human reaction (Adner et al. 2020). In a 2011 in vivo study, twenty female Hartley guinea pigs were aerosol-infected with Mtb (CDC1551) via Madison chamber to target the lungs (Thayil et al. 2011). Thayil and colleagues reported a significant increase in granuloma formation, indicative of local inflammation, within the highly vascularised choroid following exposure to ~ 200 tubercle bacilli (Thayil et al. 2011). From as early as 28 DPI, 50% of animals were found to be culture-positive for ocular Mtb infection, and by day 56 all animals were positive for infection, yielding bacterial loads ranging between 10^2^ and 10^3^ per eye (Thayil et al. 2011). Inflammatory responses were paralleled between the lung and eye with prominent immunohistochemical vascular endothelial growth factor (VEGF) staining detected around granulomatous lesions in exposed lungs as well as the retinal pigment epithelium of the eye as early as day 28, and reaching photoreceptor outer segments by day 56, no detection was found in control lung or eyes (Thayil et al. 2011). Pimonidazole staining was also consistent between both systems, with hypoxia detected in granulomas of both the lung and the choroid by day 56, indicating potential systemic immunological crosstalk, potentially through the bloodstream (Thayil et al. 2011). Fifty six DPI, a loss of vascularisation was found in 75% of animals, evidenced by retinal imaging revealing a decrease in choroidal vasculature and localised bleeding in the choroidal vessels into the choroid and retinal tissue (Thayil et al. 2011). Hematogenous seeding was hence put forth as the mechanism for translocation of Mtb bacilli from the respiratory system to the eye, a suggestion reinforced by a lack of classical afferent lymphatics to the choroid (Thayil et al. 2011; Yan et al. 2025). This idea was further substantiated through culture-based detection of bacilli in the highly vascularised spleen in 75% of infected animals by day 28, concurrent with the positive detection in the ocular tissues, challenging the possibility of lymphatic transport alone due to a lack of afferent lymphatic vessels in the spleen in both humans and rodents (Lewis et al. 2019; Thayil et al. 2011).

The potential involvement of a lung–eye axis in multiple strains of Mtb translocation has been showcased throughout various animal models and forms of infection. Abhishek and colleagues intravenously injected mice with the H37Rv strain of Mtb (Abhishek et al. 2019). The study found that 45% of infected mice had microbiologically detectable colony forming units (CFU) isolated from the eyes; moreover, 100% of mice had bacterial RNA detectable in eye lysates, exhibiting a bacterial presence (Abhishek et al. 2019). Although not all mice had detectable CFU, the presence of Mtb RNA in ocular lysates indicates ongoing gene expression and potential metabolic activity, supporting the infection at a molecular level within the eye. Hematogenous spread was evidenced by the model’s intravenous infection design, of which yielded substantial bacterial loads in the lungs and spleen, both of which measured up to 10^6^ CFU (Abhishek et al. 2019). The discovery of Mtb in the spleen concurrently with high measurements in the lung is an important observation due to the spleen receiving and filtering blood only, and thus further evidencing the hematogenous route of Mtb spread (Lewis et al. 2019).

Of importance, microarray analysis of the transcriptome of the infected eye detected 12 Mtb genes, five of which (*Rv0962c, Rv0984, Rv2612c, Rv0974c,* and *Rv0971c*), have been identified in human cases of intraocular tuberculosis (Abdisamadov & Tursunov 2020; Abhishek et al. 2019). The consistent upregulation of these genes in both mice and humans proposes their significant involvement in the ability of Mtb to invade and replicate in the human ocular system (Abhishek et al. 2019). The genes belong to the in vivo*-*expressed genomic island region, a region expressed solely during bacterial infection, as corroborated by other studies (Saw et al. 2016), and may provide a potential biomarker and possible therapeutic target due to the high conservation of genes and minimal genome variation between Mtb strains (Dar et al. 2020).

The potential implications of the lung–eye axis across species and strains of tuberculosis have been inadvertently observed for over 100 years, with the first recorded in 1899, where systemic changes were studied in animals following infection of living bacilli into the arterial circulation (Finnoff 1924). A follow-on study further elucidated bacterial dissemination mechanisms via the injection of both live and dead (heat inactivated) *M. bovis* clumps into the common carotid arteries of rabbits (Finnoff 1924; Olea-Popelka et al. 2017). *Mycobacterium bovis,* a strain known to cross the species barrier and cause zoonotic tuberculosis in humans, belongs to the Mtb complex and shares over 99% of DNA, thus providing valuable insight into possible crosstalk of bacterial dissemination across the ocular and respiratory systems (Zimpel et al. 2017).

**Fig. 2:**
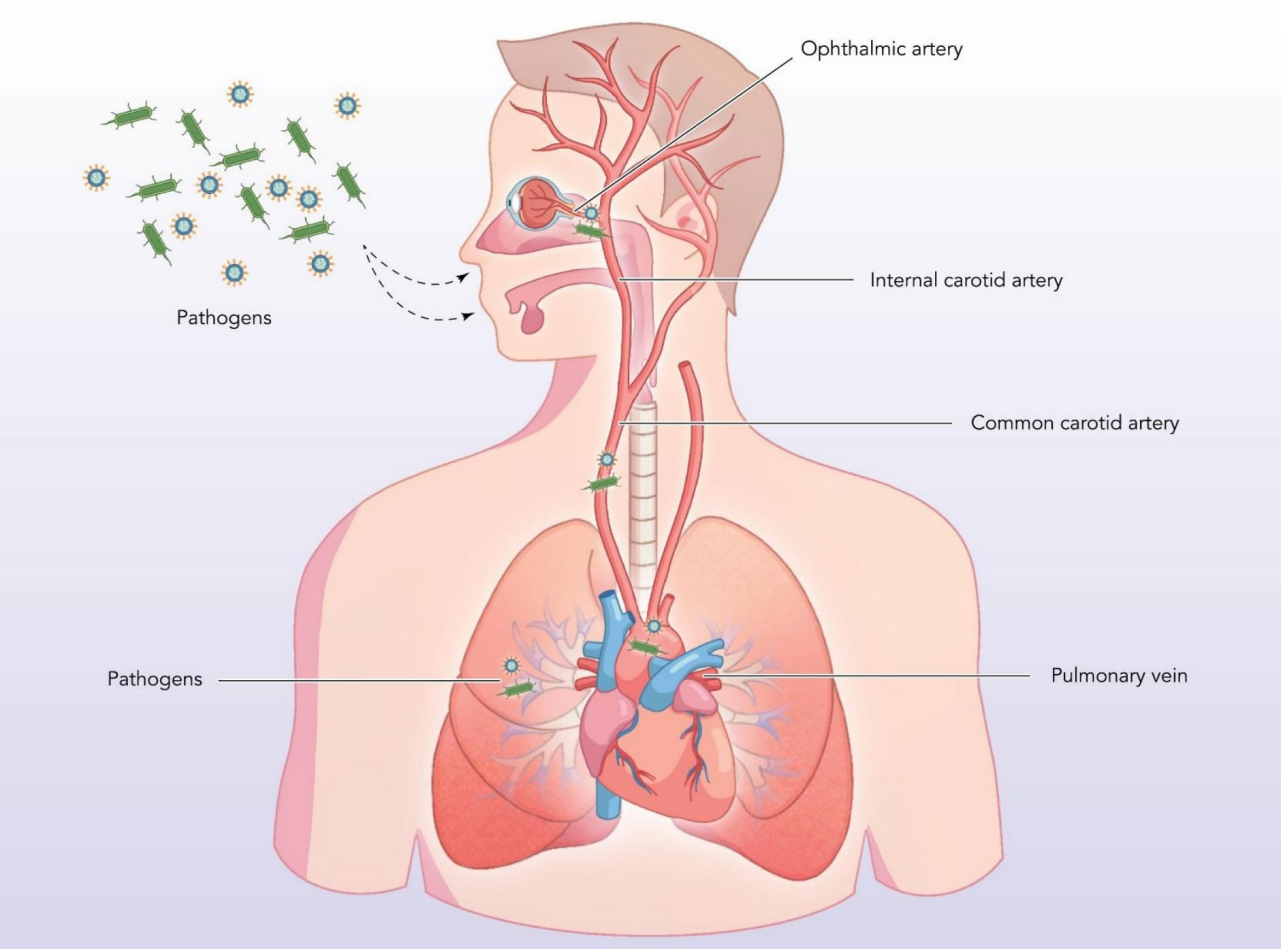
Ocular and respiratory system vascularity within the lung–eye axis. Bacteria and viruses, among other pathogens, can invade the respiratory system through inhalation, allowing for lung infection. Pathogenic transmission to the ocular system can arise through hematogenic dissemination via the common carotid artery and its subsequent branches, the internal carotid artery and the ophthalmic artery, resulting in invasion. The above schematic illustrates the physiological connection linking the ocular and respiratory systems, highlighting potential passage for distant organ immune communication and inflammation

Seventy percent of the rabbits administered with the dead bacterium exhibited ocular inflammation, and 100% of the rabbits injected with the live bacterium developed ocular inflammation (Finnoff 1924). The formation of granulomatous lesions was found in the iris, cornea, conjunctiva, and choroid along with inflammation in these regions 14 DPI (Finnoff 1924). It was noted that choroidal infiltration occurred within the first week after infection, supporting the present hypothesis of hematogenous spread due to the high vascularisation of the choroid, and the blood supplementation of the eye through the common carotid artery (Fig. 2) (Charlick & Das 2025; Finnoff 1924). Despite the paper being over a century old at present, the study and experimental methods have been validated in recent times and has corroborated that even dead bacilli within the ocular environment is able to induce detectable inflammatory responses, postulating the possibility of systemic priming from a lung infection can influence immunology of the ocular system (Charlick & Das 2025).

More recently, other animal models, such as the zebrafish model, have been used to study the spread of other bacteria strains outside of the mycobacterium tuberculosis complex to the ocular system (Takaki et al. 2018). Takaki and colleagues used *M. marinum* to infect zebrafish larva, a species chosen due to the development of the eye, structurally comparable ocular system with endothelial and retinal pigment epithelium blood retinal barriers (BRB) similar to humans, allowing the results to specifically investigate the impact of the BRB, of which was yet to be determined (Takaki et al. 2018). Furthermore, zebrafish offer an innate immune system comparable to that of humans, including granuloma formation, macrophage and neutrophil recognition and downstream cytokine (TNFα and IL-1) driven responses (Myllymäki et al. 2016). 4The study further confirmed the hematogenous spread and crossing of the blood retinal barrier, demonstrating lack of inhibition by the barrier and continued seeding of ocular mycobacteria in ~ 20% of their samples (Takaki et al. 2018). Of importance, this discovery suggested that ocular infections may result from a resolved tuberculosis infection from elsewhere in the body that has seeded in the eye. Such a mechanism may explain the ~ 50% of individuals with ocular tuberculosis yet no systemic presentation (Krishnan et al. 2010; Takaki et al. 2018; Wroblewski et al. 2011).

Bacterial transmission from the lung to the eye is not only possible but has been documented in various animal studies (Mathis et al. 2019). The identification of animal models, including zebrafish, mice and rabbits, emphasize the role of vascular dissemination in the link between the ocular and respiratory system by way of *Mycobacterium* movement and may apply to other bacterial species. The findings of acid-fast organisms, bacterial DNA and RNA in the ocular region provides evidence of systemic dissemination of bacterial disease. Importantly, with the identification of the genomic island also associated in the human disease, the studies suggest a conserved pathophysiological dynamic in the disease across species. The lung–eye axis is further affirmed as a pathway for systemic spread and opens the door for further exploration among other bacterial species.

## Cancer metastasis

Despite minimal literature exploring a connection between the ocular and respiratory system in cancer, the proposal has been around for over 100 years, tracing back to the nineteenth century and built upon in 1926. Tooker assumed that carcinomas in the choroid and iris are secondary, with documented primary carcinoma arising from other organs, namely, the breast and lung (Tooker 1926). Tooker observed tumour emboli within the anterior portion of the long posterior ciliary artery, arising from the ophthalmic artery, resulting from metastatic breast cancer of a patient, suggesting the involvement of systemic circulation in the cancer movement (Tooker 1926). Despite the observation occurring in metastatic breast cancer, the finding highlights the potential of hematogenous spread to the ocular system and is one of the first to suggest the applicability of such metastasis from the lungs (Tooker 1926). Due to the high vascularisation of eye tissues, particularly the choroid and conjunctiva, ocular tumour metastasis is not only plausible but has been documented in recent articles and case studies (Karunanithi et al. 2014). Lung cancer has become the leading cause of cancer-related deaths, with frequent metastatic propensity, and has now been recorded to affect the ocular system (Kratzer et al. 2024).

A 142-patient evaluation study of uveal (choroid, ciliary body and iris) metastasis concluded that lung cancer was the most common primary tumour site, accounting for 35% of cases (Shields et al. 1997). Ocular metastasis has been reported in the choroid, iris, ciliary body, orbit, retina, optic nerve, and conjunctiva (Yan et al. 2017). However, despite the vast number of ocular regions susceptible to metastasis due to blood flow, up to 23% of patients are asymptomatic (Yan et al. 2017). The importance of such diagnoses cannot be undervalued and opens the possibility of ocular screening for the identification of cancer severity, progression and potentially metastasis patterns.

### Non-small cell lung cancer and the choroid

The choroid, being one of the highest receivers of blood, has a relatively high incidence of non-small cell lung cancer metastasis from the lung (NSCLC) (Riess et al. 2013). A 2014 case report found the documented occurrence of choroidal metastasis from lung cancer to be ~ 9% (Riess et al. 2013). A 59-year-old female, diagnosed with stage IV lung adenocarcinoma, exhibited a lesion on her left eye following 14 cycles of systemic chemotherapy (Namad et al. 2014). A chest scan revealed an increased size of lung nodules despite normal oxygen saturation. Multiple choroidal infiltrating tumours were found and determined consistent with metastasis from the lung, resulting in the patient passing mere weeks following the discovery (Namad et al. 2014; Qu et al. 2021a, b). Despite simple diagnosis, the asymptomatic nature of such metastases can result in a miscalculated prevalence and may account for ~ 30% of choroidal metastasis (Namad et al. 2014). Presenting symptoms included blurry vision, pain, photopsia, red eye and/or a loss of vision regardless of macular appearance (Namad et al. 2014). While no specific ocular treatments exist for such instances, approaches for management include radiotherapy treatments, surgical removal, systemic chemotherapy or transpupillary thermotherapy (Namad et al. 2014).

Due to gaps in the literature, no definitive mechanism has been established, yet hypotheses of metastasis have been suggested. The anatomical–mechanical mechanism suggests probable involvement of the vascular system to the choroid, yet this is discredited due to the lack of afferent lymphatics vessels in the choroid and points to hematogenous dissemination as the primary mechanism (Mathis et al. 2019; Nema & Nema 2021).

The posterior ciliary arteries, a branch of the ophthalmic artery, provide abundant blood supply to the choroid and offer access of tumour emboli to capillary beds within the ocular structure (Fig. 2) (Nema & Nema 2021; Qu et al. 2021a, b).

Paget’s “seed and soil” theory for metastatic cancer advocates that tumour cells demonstrate preferences when metastasizing, determined by compatibility of the tumour emboli (seed) and microenvironment (soil) (Akhtar et al. 2019). The ecosystem of the ocular microenvironment may be suitable for tumour emboli due to the presence of various immunosuppressive factors, which limit or suppressive inflammation, such as transforming growth factor beta (TGF-b), alpha-melanocyte-stimulating hormone (a-MSH), vasoactive intestinal peptide (VIP) and indoleamine 2,3-dioxygenase (IDO), of which may offer immune detection avoidance (Qu et al. 2021a, b). Adding to immune evasion, the cornea, iris and retinal cells present immunosuppressive ligands, programmed death-ligand 1 (PD-L1) and Fas ligand (FasL), resulting in the inhibition of T cell activation and T cell apoptosis (FasL) (Qu et al. 2021a, b).

Finally, arterial infiltration of circulating tumours promotes emboli access to the “soil” via the loosely attached capillary endothelial cells in the choroid, as represented in ocular metastasis of squamous cell lung cancer, where the microenvironment of the iris allows for successful seeding of tumour cells despite comparably less blood flow than the choroid (Hiraki et al. 1998; Qu et al. 2021a, b). Choroidal endothelium is lined with small pores, increasing permeation of circulating “seeds” into choroidal tissue and allowing metastatic sites to be established, particularly due to the common adherence of malignant cells to choroidal tissue (Qu et al. 2021a, b). An important consideration is the predominance of the left eye to be targeted, understood by Stephens and Shields to be a result of 90 degree angles of the internal carotid artery and ophthalmic artery, causing a reduction in blood flow velocity to the right eye (Stephens & Shields 1979).

### Lung cancer and the ocular surface

While choroidal metastasis is more frequently documented, the anterior segment, including the conjunctiva of the eye, has also been subjected to lung carcinoma metastasis. The mechanism of hematogenous spread is supported by the higher incidence of choroidal metastasis, a region with rich blood supply, compared to the anteriorly located conjunctiva, ciliary body, and iris (Karunanithi et al. 2014). Tumour clusters have been described, by Tooker, within the long and short posterior ciliary artery and the anterior artery, with these blood vessels arising from the ophthalmic artery, the primary supplier of the ocular surface (Tooker 1926). Importantly, no presence of tumour nodules was documented within the thoracic lymph glands. Although descriptions of emboli movement through the arteries have been indicated, it is important to note a lack of consistent findings amongst reports, prompting the essential need for further investigations of hematogenic and lymphatic pathways (Fig. 2).

In another study, a 50-year-old male was found with a vascularised nodule on the palpebral conjunctiva and diagnosed with highly aggressive stage IV NSCLC (Chew et al. 2014). A similar case detailed a 75-year-old man with a lower left eyelid lesion with ulceration through the conjunctiva (Joseph et al. 2016). A biopsy of the lesion discovered infiltrating adenocarcinoma and the patient was again diagnosed with stage IV NSCLC (Joseph et al. 2016). These cases demonstrate the need for ocular screening with the acknowledgement of respiratory cancer metastasis and highlight a need for more research behind the mechanisms of metastasis. Considering that a significant proportion of these cases are asymptomatic, future research should attempt to establish biomarkers for early detection.

### Immune system crosstalk in cancer metastasis

With immunology at the forefront of the lung–eye axis, the complex interplay of these immune systems may very well extend to cancer-associated diseases that cross into functionally distinct systems. The eye is home to an immune privileged site, consisting of tight junction barriers creating a blood–retinal barrier (BRB), and vascular endothelium, along with immunosuppressive factors, supporting a sanctuary resistance to immune threats in systemic circulation (Qu et al. 2021a, b; Takaki et al. 2018). However, the neuroendocrine characteristics of small cell lung cancer (SCLC) enable the breaching of the immune privileged site and over-riding peripheral tolerance within the retina, through cell type recognition in the retina, and subsequently manifesting ophthalmological diseases such as autoimmune retinopathy (Bazhin et al. 2007; Lu et al. 2010). These diseases, namely, cancer-associated retinopathy (CAR), are caused by lung cancer cells, particularly SCLC, cells expressing retina specific antigen, recoverin, being released into circulation, eliciting a high-affinity anti-recoverin autoantibody into the blood stream as a response, compromising the BRB (Bazhin et al. 2007) It has been evidenced that 15% of SCLC patient have both detectable recoverin autoantibodies in serum, prior to the clinical signs of CAR (Bazhin et al. 2007). This offers several crucial insights regarding immune crosstalk and antigen translocation, implying that antigenic components from lung cancer tumours may elicit immune responses, even in the immune privileged eye, without migration of tumour emboli itself. Another important aspect is the ability of peripheral tumours to behave as a molecular mimic to prime the niche environment of the ocular immune system. It has been postulated that autoimmune reactions may direct the cancer-induced anti-recoverin autoantibodies to the retina and subsequently bind recoverin in photoreceptors, triggering inflammation and cell death (Matsubara et al. 1996). After incubating a normal rat retina with section with a CAR-positive patient’s serum, that contained autoantibodies against recoverin, the serum specifically bound to the photoreceptor layer within the retina, the location of recoverin (Matsubara et al. 1996). Importantly, this highlights the potential for the autoantibodies released in systemic circulation to indeed bind within the ocular environment, a control serum showed no binding within the ocular environment (Matsubara et al. 1996).

Concurrently, dysbiosis in the lung microbiome involvement has been suggested in immune modulation and tumour migration to distant sites, including the eye. A KP mouse model of lung cancer was infected with *Veiollena parvula*, a micro-organism frequent in human lung cancer, to induce dysbiosis in the lower respiratory system and the paraneoplastic markers were observed (Tsay et al. 2021). The enrichment of the bacterium resulted in accelerated tumour growth, upregulation of immune checkpoint markers, PD-1, and an increase in inflammatory cytokines, such as IL-17 and PI3K/AKT signalling pathways, revealing a pro-metastatic immune environment (Tsay et al. 2021). Furthermore, systemic alterations in microbiome as a result of lung cancer has been found with increases of *Prevotella* (*P* = 0.0044) and *Veillonella* (*P* = 0.02), correlating with higher systemic immune-inflammation index (Cheng & Wang 2025) Immune suppression was also suggested through a reduction in CD8 + systemically (*P* = 0.0027) (Cheng & Wang 2025). Taken together, these results imply that lung microbiota-induced immune suppression is not only possible but contributes to tumour growth, progression and has the potential to trigger autoimmune responses in distant locations, potentially over-riding the immune privileged environment of the eye.

### Lung cancer metastasis from the eye

It is important to recognize the potential for bidirectionality of metastatic spread of cancers between the ocular and the respiratory systems. Rishi et al*.* used histopathological analysis to detect mucoepidermoid carcinoma originating from the conjunctiva, with tumour clusters found extending into orbital vascular lumen of a patient with reduced vision in the right eye (Rishi et al. 2015). Emboli were revealed to have migrated to the orbital tissue, muscle fibres, sclera, choroid, and optic nerve (Rishi et al. 2015). The patient was found to have squamous cell carcinoma in the left lung following the misdiagnosis of tuberculosis, a not uncommon occurrence (Rishi et al. 2015). While specific mechanisms of metastasis have yet to be described in the literature, Rishi and colleagues supported the notion of hematogenous spread due to the distance of metastasis and the aggression of the tumour within orbital tissue. Further clarification is required for the exact route for such metastasis to enable a better therapeutic intervention, particularly due to a 79% rate of recurrence (Rishi et al. 2015). Seitz and Henke recommend the complete removal of the eyeball as a primary treatment to circumvent metastatic invasion to systemic tissues, exemplifying the dire need for dedicated elucidation in the prospective bidirectionality between the ocular and respiratory systems to prevent such measures needing to be taken (Seitz & Henke 1995).

Robinson et al*.* reviewed 21 cases of mucoepidermoid carcinoma of the conjunctiva and found the lacrimal sac is included in the tissues affected by such disease (Robinson et al. 2006). As the lacrimal sac initiates the NLD connection to the respiratory system, the metastasis from the ocular surface to the lacrimal channel suggests a potential path for invasion of the pulmonary system (Robinson et al. 2006). This was reported by Kumar and colleagues, establishing that secondary involvement of the lacrimal sac and NLD can result from cutaneous tumour spreading involving the conjunctiva (Kumar et al. 2016). Eighty-nine percent of patients experienced tumour spread from the lacrimal sac to the medial canthus, where the eyelids meet, with another 11% having intraconal orbital spread (Kumar et al. 2016). Furthermore, 38% of nasolacrimal tumours were able to spread to the sinus or nasal cavity, indicating the potential for a tumour to arise in the conjunctiva, spread through the lacrimal sac and NLD, and enter the upper respiratory system (Kumar et al. 2016). The bidirectional activity is again postulated in this pathway, where magnetic resonance imaging demonstrated high signal intensity in a case of recurrent melanoma spreading from the nasal cavity to the NLD in one patient. In additional support, Janakiram et al*.* noted the expansion of squamous cell carcinoma from the lacrimal sac to detection within the NLD (Janakiram et al. 2016).

With lung cancer becoming more prevalent in recent times, the ocular implications are becoming vital to comprehend for potential biomarkers and metastatic activity. A clear translational gap exists between the clinical manifestations of ocular metastasis in patients and the ability of experimental models to replicate the process, highlighting a need for more robust approaches and investigation. Such models could utilise zebrafish with transparent architecture and human-like ocular structures to follow cancer cell circulation. Furthermore, it has been substantiated that a lack of understanding about said pathways has yielded faster mortality rates and unsuitable treatment options. Being that metastasis to the eye is an initial symptom of advanced lung cancer, improving understanding of this area can lead to earlier detection and more appropriate planning. Finally, monitoring of the ocular tissues, should be implemented to cancer observations for both clinicians and patients for improving prognosis.

## Glaucoma and chronic lung diseases

### Glaucoma

**Fig. 3:**
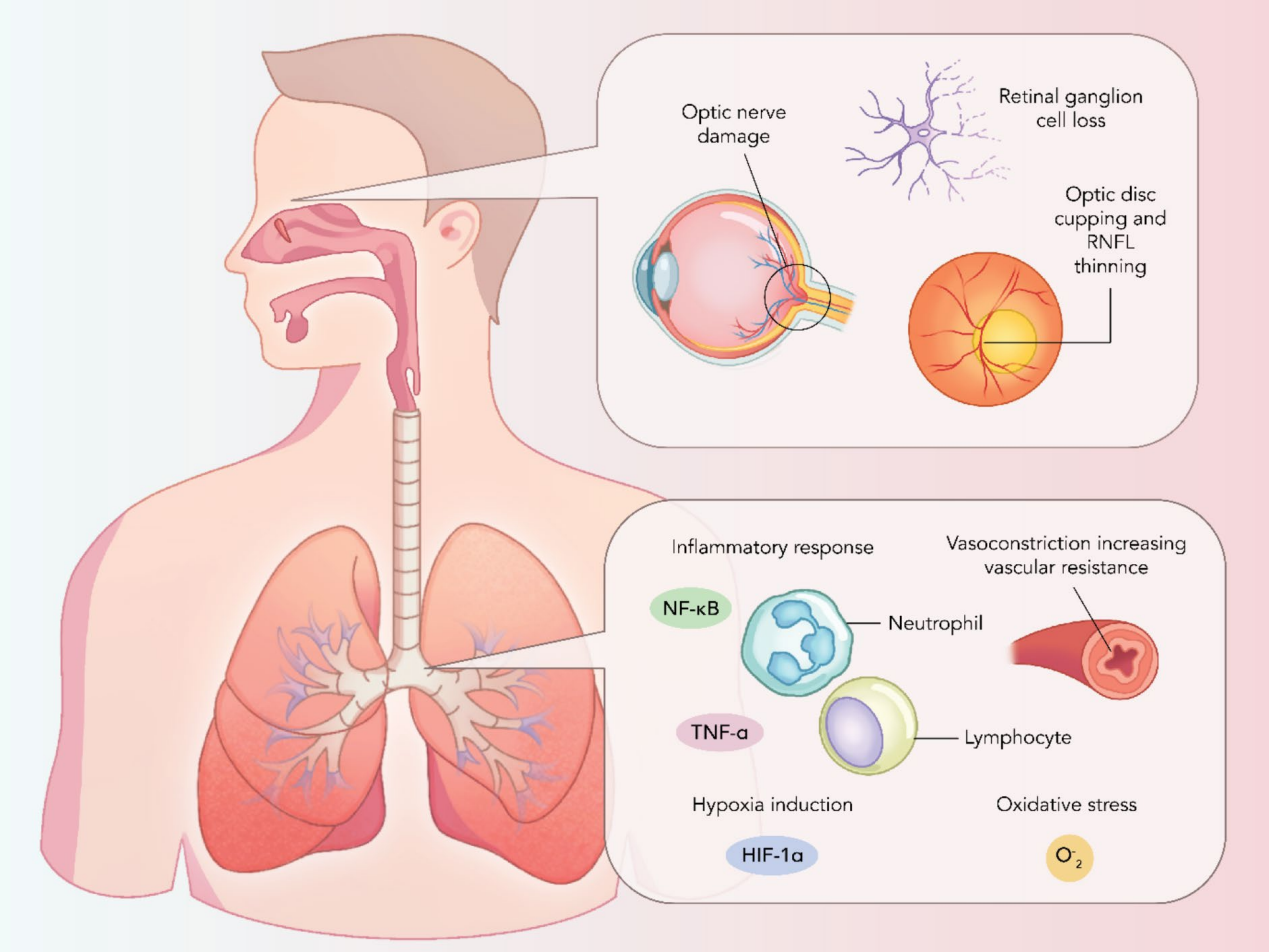
Chronic respiratory disease impact on glaucoma pathology. This figure highlights the potential communication between the ocular and respiratory systems, accentuating the impact of hypoxia induction (HIF-1a), oxidative stress (O-2) and inflammation in disease acceleration and pathogenesis. Key inflammatory signalling molecules, including NF-kB and TNF-a, are emphasized, prompting their role in inflammatory responses, including neutrophil and lymphocyte activation, culminating in vasoconstriction along with hypoxia-induced mechanisms. Ocular implications, encompassing optic nerve damage, retinal ganglion cell depletion, optic disc cupping and retinal nerve fibre layer (RNFL) thinning, are frequently noted in illnesses related to systemic hypoxia and inflammation of which is prevalent in COPD and OSA. The mechanisms observed postulate the existence of a bidirectional relationship between the lung and eye, with manifestations of inflammation and oxygen deficiency conducing to ocular and pulmonary pathophysiology

Glaucoma is the primary cause of irrevocable blindness across the world, with more than a 31% increase in incidence between 2010 and 2020 and impacting up to 79.6 million individuals in 2020 (Flaxman et al. 2017; Hsu & Desai 2023). The hallmark characteristic of the disease is the deterioration of retinal ganglion cells (RGCs), resulting in optic nerve damage, visual field deficit, and culminating in permanent loss of vision (Shen et al. 2021) (Fig. 3). Retinal ganglion cells sit within the inner surface of the eye and its axons compose the retinal nerve fibre layer (RNFL), which converges at the optic disc, forming the optic nerve (Ventura et al. 2006). While not definitive, the current literature has focussed on elevated intraocular pressure (IOP) as the primary risk factor, where there is an imbalance between the production and outflow of the nourishing aqueous humour (Hsu & Desai 2023). Because of this, all therapeutic options at present attempt to use pharmacological or surgical intervention to lower IOP, yet this only slows disease progression. The neurodegenerative disease has two main subtypes: open-angle glaucoma (OAG) and angle-closure glaucoma (ACG), both of which have been identified with elevated IOP (Hsu & Desai 2023). Other subtypes include primary open-angle glaucoma (POAG), the most common subtype, normal tension glaucoma (NTG), which lacks elevated IOP (Killer & Pircher 2018), congenital glaucoma, pigmentary glaucoma, and traumatic glaucoma (Shen et al. 2021).

### Glaucoma and COPD

Due to a lack of documented systemic impact in glaucoma, pulmonary function has sustained little consideration in its pathogenesis. Chronic obstructive pulmonary disease (COPD) has a high global prevalence with a rising trend and system-wide repercussions that include systemic inflammation, hypoxia, oxidative stress, and vascular dysfunction (Nucera et al. 2022; Ugurlu et al. 2018). The disease has been associated with changes to the small blood vessels of the ocular system via chronic hypoxia, evidenced by the overexpression of hypoxia-inducible factor (HIF)−1α in COPD lungs (Shukla et al. 2020), and inflammatory invasion (Singh et al. 2024). Such inflammation has been suggested to extend systemically, resulting in widespread physiological impact away from the lungs, evidenced by upregulations of IL-6, IL-8, TNF-α, fibrinogen, white blood cells, and C-reactive protein in circulation in COPD patients (Agustí et al. 2012). Augsti et al*.* further found that 70% of patients with COPD had some constituent of systemic inflammation and 16% had continuous systemic inflammation, of which was associated with greater mortality and increased frequency (Agustí et al. 2012).

Ocular structures, including the choroid, RNFL, and retinal vascular vessels, are suspected to be affected by the pulmonary disease as evidenced by Ozcimen and colleagues (Ozcimen et al. 2016). The study applied optical coherence tomography (OCT) to establish the thinning of the RNFL and peripapillary choroid in COPD, both of which have been suggested in glaucoma pathogenesis (Lin et al. 2016; Ozcimen et al. 2016). A correlation was found between the RNFL thickness and peripapillary choroidal thickness in COPD patients; however, this was not reflected in the controls, suggesting specific pathological changes occurring in association with impaired pulmonary function in COPD, supporting the existence of a lung–eye axis (Ozcimen et al. 2016). Importantly, the authors state the increased susceptibility of choroidal and retinal structures to ocular diseases due to the optic disc reduction through RNFL thinning (Ozcimen et al. 2016). Here, we see how degenerative diseases such as glaucoma become a possibility, particularly through the increase of vascular resistance, making structures more vulnerable to pulmonary-stimulated hypoxia (Ozcimen et al. 2016).

Ugurlu et al*.* observed an increasing diameter of retinal veins and a thinning of the circumpapillary RNFL and assumed relation to COPD-induced hypoxia (Ugurlu et al. 2018). A cross-sectional study including 104 glaucomatous patients and 59 healthy patients demonstrated a correlation between the RNFL thickness and glaucoma severity via the visual field index (Kang et al. 2015). The inferior RNFL quadrant had the strongest association with visual field index, a quadrant evidenced to be thinner in individuals with COPD, irrespective of the severity (Kang et al. 2015; Wagh et al. 2022). Such results were again detected in a 2024 meta-analysis, concluding a statistically significant reduction in superior and inferior RNFL’s in COPD patients when compared with healthy controls (Kazantzis et al. 2024). The thinning of the RNFL has been argued as a better diagnostic than IOP due to the latter’s elevation present in healthy individuals with no signs of glaucoma (Pfeiffer et al. 2002). As such, the systemic pathological mechanisms of COPD, including chronic inflammation, hypoxia, vascular dysfunction and oxidative stress (Ugurlu et al. 2018), not only postulate that pulmonary function may relate to ocular health, but may also independently contribute to glaucoma pathogenesis via mechanisms not yet fully understood.

A 2022 study from Korea investigated the presence of OAG in female with COPD against females with normal pulmonary performance (Lee et al. 2022). The study utilised a National Health Survey between 2008 and 2011 with a total of 5039 females under 40 years and employed a cross-sectional analysis. The resulting trend suggested that as pulmonary function worsens in females, glaucoma incidence increases, demonstrated by restrictive function having higher glaucoma presence than normal (6.4% vs 4.8%). Furthermore, a significant difference (*p* < 0.001) was detected, indicating a higher likelihood of glaucoma in females with COPD compared to females with normal pulmonary function (Lee et al. 2022).

A possible sex difference may be highlighted in the pathogenesis of glaucoma and is further supported when considering the divergence of disease courses compared to males. In COPD, females tend to be subjected to higher recurrence of exacerbations, greater airway inflammation and expression of pro-inflammatory cytokines such as IL-17 (Torres et al. 2007, 2011; Gut-Gobert et al. 2019). In addition, it was found that non-smoking COPD patients had further increased risk of OAG development, possibly due to the higher rates of shortness of breath and lower arterial oxygen saturation compared smoking-induced COPD (Meneghini et al. 2019). The latter is also associated with RGCs loss and should be a consideration in other respiratory conditions such as obstructive sleep apnoea (Lee et al. 2022; Meneghini et al. 2019; Sivakumar et al. 2013). Finally, the study called to question IOP being a primary risk factor to OAG, with IOP values significantly lower in COPD–glaucomatous individuals than glaucomatous individuals alone, suggesting the possibility of pulmonary function as an independent risk factor for OAG (Lee et al. 2022).

To summarise, the above section offers evidence of COPD-related mechanisms influencing glaucoma progression through the induced hypoxia and its downstream effects. Furthermore, this section has demonstrated the involvement of hypoxia and oxidative stress on the hallmark characteristic, RGC loss, in the absence of elevated IOP. Despite early stages of the above literature, numerous occasions of a relationship have been demonstrated with pathogenesis separate from IOP and in multiple forms of the degenerative disease. Finally, differences not yet explored have been noted in glaucomatous patients with COPD, including sex implications, and offer new routes of investigation for a better understanding of both diseases and the interplay between them. Importantly, the lung–eye axis becomes more prevalent when considering the correlation between hypoxia-induced mechanisms and structural changes present in jeopardised lung function, as mentioned earlier. Finally, the evidence presents a new scope, viewing altered pulmonary function as a risk factor for glaucoma, pushing future research to further investigate this relationship.

### Obstructive sleep apnea (OSA) and glaucoma

Obstructive sleep apnea (OSA) is a chronic respiratory disease, existing under the umbrella term of sleep apnea, and is one of the most ubiquitous respiratory diseases in patient care (McNicholas 2009). The following section includes literature encompassing conditions of “obstructive sleep apnea”, “obstructive sleep apnea hypopnea syndrome”, and “sleep apnea syndrome” and uses these terms interchangeably if the conditions have the same pulmonary implications and pathology. Characteristics of OSA include recurrent occurrences of upper airway obstruction, inflammation and intermittent spikes of hypoxia (Faridi et al. 2012). Current studies have demonstrated OSA-stimulated inflammatory factors, including hypoxia inducible factor (HIF-1α), NF-κB, TNF-α, inducible NO synthase (iNOS), VEGF, and cleaved caspase 3 reaching the lung and liver tissues (Rosa et al. 2012). Reductions in lung volumes have been documented in relation to disease severity, demonstrating the full respiratory system impact of the disease (Stadler et al. 2010). Prior studies have revealed ocular disorders in connection with OSA, including but not limited to floppy eye syndrome (Robert et al. 1997), keratoconus (Naderan et al. 2015), papilledema (Purvin et al. 2000), and optic neuropathy (Mojon et al. 1998). Sagir et al*.* noted a significantly thinner sub foveal choroid as well as significant reductions in both in superficial and deep retinal vessel densities in patients with severe OSA**,** suggesting a reduction in blood supply to the RGCs (Sagir et al. 2024). Such results were attributed to increases in inflammatory factors HIF-1α and TNF-α in serum, both of which are activated under the hypoxic conditions of OSA and were upregulated regardless of disease severity (Sagir et al. 2024).

With an ocular connection established, hypotheses have postulated implications of the upper respiratory disease in glaucoma pathogenesis. Walsh and Montplaisir initially proposed a possible relationship, noting more severe glaucoma correlated with more, and longer, episodes of sleep apnoea in the same generation (Walsh & Montplaisir 1982). Mojon and colleagues were then the first to quantitatively suggest that OSA patients had a significantly higher likelihood to be diagnosed with glaucoma compared to non-OSA patients (Mojon et al. 1999). The study detected positive correlations between the respiratory disturbance index, glaucomatous optic disc changes, and visual field loss (Mojon et al. 1999). The authors proposed that OSA may cause damage to the optic nerve head via the impairment of blood flow, resulting from the repetitive and prolonged episodes of hypoxia (Mojon et al. 1999). This was attributed to the potential of an imbalance between nitric oxide and endothelin, associated with hypoxic conditions, resulting in insufficient blood flow to the optic nerve, causing direct damage (Mojon et al. 1999).

Similar to COPD–glaucoma implications, RNFL thinning has been observed in OSA patients and has resulted in glaucomatous symptoms. Lin and colleagues undertook an investigation comparing RNFL thickness and optic nerve head measurements in OSA/hypopnea patients (Lin et al. 2011). Results from 127 subjects demonstrated significant (*p* < 0.0001) decreases in RNFL thickness as disease severity increased, thus resulting in the conclusion that moderate/severe OSA patients are at an elevated risk of glaucoma (Lin et al. 2011).

Furthering the pathogenic role of OSA in glaucoma, Yamada et al*.* used peripheral blood samples from 166 patients; 42 control, 109 OAG only, and 15 OAG with OSA, including NTG and POAG (Yamada et al. 2018). Measuring systemic oxidative stress via reactive oxygen metabolites, statistically higher levels of oxidative stress occurred in glaucoma–OSA patients compared to glaucoma-only patients, demonstrating pulmonary impact (Yamada et al. 2018). The functional effect of OSA was compared between groups by measuring visual field progression with underlying variables nullified, including age, sex, diabetes, smoking habits and hyperlipidaemia. Mean deviation slope of this measurement revealed a significantly steeper slope in OAG patients with OSA, marking faster visual field defect progression compared to glaucoma alone (Yamada et al. 2018). Yamada and colleagues were the first to detect visual field defect progression in glaucomatous OSA patients, a key parameter of glaucoma progression (Forchheimer et al. 2011; Yamada et al. 2018). By doing so, the study elucidated mechanisms for accelerated glaucomatous manifestations in OSA. Results from Yamada et al*.* are supported by correlative evidence of RNFL thinning (Fan et al. 2019). This was demonstrated by 64.7% glaucoma patients, with moderate/severe OSA, having significant progression of RNFL thinning, indicating a glaucomatous-structural change (Fan et al. 2019). In distinction, only 26.7% of glaucoma patients with no/mild OSA had such thinning, suggesting a pulmonary-specific effect in the ocular disease. The study concluded that severe OSA, combined with glaucoma, had a significantly higher chance of glaucomatous structural declination compared with glaucoma alone (Fan et al. 2019).

The implication of the chronic upper respiratory disease, OSA, has demonstrated mechanisms which can cause glaucomatous characteristics to worsen. Both structural and functional evidence exists and thereby implicates the role of the respiratory system in the health of the ocular system. The lung–eye axis is supported by the above section through the inflammatory markers, structural, and functional alterations induced by the respiratory implications. Such changes have included those directly afflicted in the degenerative disease, glaucoma, encompassing RGC loss, thinning of the RNFL and compromised oxygen supplementation, all of which have been evidenced apparent in OSA.

## Endothelin-1 and the lung–eye axis

Endothelin-1 (ET-1) has been observed to be elevated both in the pulmonary system and in systemic circulation of patients with chronic respiratory diseases, namely, COPD and OSA. The vasoconstrictor protein is secreted by epithelial cells in both the airway and ocular system and has been notably elevated through prolonged hypoxia, as seen in COPD and OSA, resulting in vascular alterations (Nikolaou et al. 2003; Ozcimen et al. 2016; Salvatore & Vingolo 2010; Schoen et al. 2017). With systemic increases of ET-1 resulting in significant vasoconstriction of ocular vessels, and subsequent reduction in optic-nerve head perfusion, the observed increases in respiratory diseases, COPD and OSA, may pose a risk to the ocular system of individuals. In OSA, recurrent hypoxia is experienced, driving the ET-1 release through HIF-1a signalling and oxidative stress, culminating in a 50% rise in ET-1 within the plasma (Gjørup et al. 2007). Similarly, acute COPD exacerbations increase both sputum and plasma ET-1 levels, resulting in a feedback loop of increased hypoxia generating more ET-1 (Roland et al. 2001). Considering the extended half-life of ET-1 when bound to its receptors, increases in systemic ET-1 could reach the ocular system, evidenced by ET-1 aqueous humour and plasma levels being significantly higher (*P* < 0.001) in glaucomatous patients compared to controlled (Lampsas et al. 2024). Elevation of the vasoconstrictive peptide has been implicated in numerous ocular diseases with vascular involvement, including glaucoma, diabetic retinopathy and HIV-related retinopathy yet, such fields are still in initial stages and further investigation is required (Fuchsjäger-Mayrl et al. 2003). Such involvement brings about discussion of the systemic implications of chronic respiratory disease-induced hypoxia and its impact on far-reaching systems with altered lung function impacting systemic circulation and, subsequently ocular blood flow (Okuno et al. 2006).

### Mechanism of endothelin-1

Being the most potent vasoconstrictor known, ET-1 advances the degeneration of axons of the optic nerve via vascular dysregulation, and activates apoptosis in RGCs, with their axons predominantly composing the RNFL, resulting in thinning of the layer (Kazantzis et al. 2024; Nagata et al. 2014). Human and animal studies have demonstrated the ramifications of ET-1 elevation, noting the binding of the peptide to the ET_A_ receptor on both smooth muscle and endothelial cells in the choroid and retina, subsequently constricting ocular blood flow (Salvatore & Vingolo 2010). Vascular endothelial cells are the primary secretors of ET-1 in the eye allowing for irreversible binding to the ET_A_ receptors in the choroid and retina vascular smooth muscle cells, resulting in reduced blood flow in these tissues for long-lasting suppression (Salvatore & Vingolo 2010). Ocular implications from systemic upregulation of ET-1 may be assumed due to the presence of both the vasoconstrictive peptide and NO in the ophthalmic artery, as demonstrated in a dysfunction of ocular endothelial cells in diabetes and hypertension (Salvatore & Vingolo 2010). Furthermore, ET-1 can be secreted by leukocytes, of which are at an increased level, in the peripheral blood of both stable COPD and OSA patients and is increased further during exacerbations (Atkeson et al. 2009; Barbu et al. 2011).

### Endothelin-1 in glaucoma pathogenesis

Despite a lack of extensive investigation, ET-1 has been implicated in the pathogenesis of varying glaucomatous subtypes, including POAG, ACG and NTG (Li et al. 2016) (Chen et al. 2013). Li et al*.* used a meta-analysis including 7 NTG studies and 6 POAG studies to determine the association between plasma ET-1 and the development of either glaucoma subtype (Li et al. 2016). Of note, this meta-analysis was the first to conduct a study regarding the relationship between ET-1 and glaucoma, illustrating the gap in chronic pulmonary diseases and glaucomatous literature. Results from the study revealed that in both NTG and POAG, the ET-1 plasma levels were elevated compared to healthy controls and advocated that ET-1 concentrations are significantly associated with POAG in particular (Li et al. 2016).

The original research by Chen et al*.* studied the relationship between elevated ET-1 levels and the RNFL in 31 primary OAG patients, 18 NTG patients, and 16 primary ACG patients (Chen et al. 2013). Using visual field testing and OCT, the ET-1 levels were determined significantly elevated compared to healthy controls in all 3 groups (Chen et al. 2013). Lakshmanan et al*.* used IV injection of ET-1 in genetically identical mice to evaluate long-term effects of elevated ET-1 (Lakshmanan et al. 2023). The experiment illustrated a long-term dose–response effect of ET-1 and the expedition in RNFL thinning, insufficient retinal blood flow, decreased retinal cell responses and RGC apoptosis (Lakshmanan et al. 2023). RGC responses were decreased, as measured by reduced b-wave, a measurement sensitive to glaucomatous damage as reductions in b-wave amplitude are indications of bipolar cell dysfunction, suggesting improper relaying of signals from photoreceptors to RGCs, resulting in overstimulation, and possible degeneration, of RGCs through excessive glutamate release (Boccuni & Fairless 2022; Lakshmanan et al. 2023; Wilsey & Fortune 2016). At day 10, all dosed groups showed a significant reduction in b-wave responses, and at day 28, had significant reductions in higher doses (Lakshmanan et al. 2023). The reduction in b-wave responses have been shown to determine the activity of the axons of RGCs, implicating the RNFL (Yang et al. 2024). With only higher concentrations demonstrating significant reductions, ET-1 is implicated in the mechanism of visual defect, as lower concentrations recovered after time, thus supporting the interconnection of chronic respiratory diseases and glaucoma with continuously elevated ET-1.

The pathophysiological connection between glaucoma, COPD, and OSA is critical for the understanding of all three diseases. The demonstrated shared risk factors of hypoxia and oxidative stress suggest an underlying link with the potential for early indications and biomarkers of glaucoma and respiratory disease management. Acknowledging the inter-relationships between these diseases can optimise treatments and understanding the fluctuations in blood vessel function, including vessel diameter and resistance to prepare better treatment options.

## Conclusions and future directions

Despite the infancy of the lung–eye axis field, implications of a connection between the respiratory and ocular systems have been around since the 1800 s, with the implication of cancer metastasis from the lung to the eye. Since its initial inception, associations have been documented throughout diseases, ranging from acute bacterial and viral infections to chronic conditions, where immune system and microbiome have been suggested to communicate and affect systemic health. The anatomical bridge, the nasolacrimal duct (NLD), has been delved into as a point of transmission for both acute and cancerous diseases, summating in evidence for pathogen transfer as well as a bidirectional pathway for malignant tumours.

This review has highlighted the process of cancer metastasis, with a focus on malignant transfer through hematogenous spread and further postulates the bidirectionality regarding transmission from the eye to the lung, with limited previous literature focusing on the unidirectional movement of lung to eye metastasis. The connection established here has previously been underexplored and prompts further investigation. Chronic respiratory diseases, including OSA and COPD, are discussed in relation to a potential sharing of pathophysiological mechanisms to chronic ocular diseases, namely, glaucoma. The frequent hypoxia amongst such diseases is characterised and, together with vascular dysfunction and increased ET-1 expression, are explored as a potential connection within worsening respiratory and eye health.

With axial networks, the eye–gut and gut–lung, gaining further traction and investigation in the scientific community, the potential influence of systemic crosstalk is becoming more prevalent. The interconnection of these axes offers a broad spectrum for future research, suggesting that human health may be far more reliant on intersystem linkages than previously thought.

With the notion of a lung–eye axis still in its inception, the future of literature should focus on closing the gaps regarding systemic interaction. The microbiome interactions between the ocular and respiratory system represent an area lacking in understanding, particularly the specific communication between mucosal immunity of which both systems have. Studies going forward should explore the ocular microbial communities, as the tissue was originally assumed devoid of bacteria, and the implications such species may have in the cross-communication of inflammatory conditions.

While the idea of ocular metastasis in cancer and bacteria has been present for over 100 years, specific mechanisms, be it hematogenous or lymphatic, are not yet known, demonstrating the need for future research to investigate biomarkers expressed in early inflammation to allow for specific monitoring and targeted therapies.

The involvement of hypoxia-induced damage has been implicated across respiratory conditions and ocular diseases, with inter-relationships between the systems progressing, and possibly accelerating, diseases. The prevalence of hypoxic pathways between these systems should be explored, allowing potential therapies, such as oxygen supplementation, to enable the extent of hypoxic involvement in the development of disease.

The introduction of the lung–eye axis has allowed for research to come to light in the impact of the respiratory system on the ocular system; however, the bidirectional relationship of the eye’s impact on the pulmonary system should be further investigated. Such investigations could yield inflammatory mediators expressed in the lungs of ocular-diseased patients, allowing for a stronger understanding of the shared pathophysiology in disease states.

Finally, long-term studies, with a focus on the crosstalk between the ocular and respiratory systems during disease progression with an emphasis on the acceleration on disease severity and pulmonary medications that have off-target ocular implications, as seen in some asthmatic medications. Such studies may offer insight into treatment plans and better mechanistic understandings of systemic communication under therapeutic intervention.

The complex field of the lung–eye axis is a new area with little understanding yet vast potential for its role in human health. The interplay between these systems highlights the idea of crosstalk throughout systems. New insights and therapeutic strategies can not only fill voids in the understanding of the ocular and respiratory system but may also elucidate similar channels of interaction between other systems. Looking across all disease states, acute, chronic, and cancerous, a relationship appears prevalent across the eye and lung and may open a new world for innovative treatments and new understanding to human health.

## Abbreviations

ACE2: Angiotensin-converting enzyme 2
COPD: Chronic obstructive pulmonary disease
ET-1: Endothelin-1
HIF-1a: Hypoxia inducible factor 1 alpha
HKU1: Human coronavirus HKU1
IL-: Interleukin-
NLD: Nasolacrimal duct
NO: Nitric oxide
NTG: Normal tension glaucoma
OAG: Open-angle glaucoma
OSA: Obstructive sleep apnea
POAG: Primary open-angle glaucoma
RGC: Retinal ganglion cell
RNFL: Retinal nerve fiber layer
RSV: Respiratory syncytial virus
RT-qPCR: Real-time quantitative polymerase chain reaction
SARS-CoV-2: Severe acute respiratory syndrome coronavirus 2
TMPRSS2: Transmembrane serine protease 2
